# Evaluation of a microarray-hybridization based method applicable for discovery of single nucleotide polymorphisms (SNPs) in the *Pseudomonas aeruginosa *genome

**DOI:** 10.1186/1471-2164-10-29

**Published:** 2009-01-19

**Authors:** Andreas Dötsch, Claudia Pommerenke, Florian Bredenbruch, Robert Geffers, Susanne Häussler

**Affiliations:** 1 Helmholtz Centre for Infection Research, Inhoffenstraße 7, 38124 Braunschweig, Germany

## Abstract

**Background:**

Whole genome sequencing techniques have added a new dimension to studies on bacterial adaptation, evolution and diversity in chronic infections. By using this powerful approach it was demonstrated that *Pseudomonas aeruginosa *undergoes intense genetic adaptation processes, crucial in the development of persistent disease. The challenge ahead is to identify universal infection relevant adaptive bacterial traits as potential targets for the development of alternative treatment strategies.

**Results:**

We developed a microarray-based method applicable for discovery of single nucleotide polymorphisms (SNPs) in *P. aeruginosa *as an easy and economical alternative to whole genome sequencing. About 50% of all SNPs theoretically covered by the array could be detected in a comparative hybridization of PAO1 and PA14 genomes at high specificity (> 0.996). Variations larger than SNPs were detected at much higher sensitivities, reaching nearly 100% for genetic differences affecting multiple consecutive probe oligonucleotides. The detailed comparison of the *in silico *alignment with experimental hybridization data lead to the identification of various factors influencing sensitivity and specificity in SNP detection and to the identification of strain specific features such as a large deletion within the PA4684 and PA4685 genes in the Washington Genome Center PAO1.

**Conclusion:**

The application of the genome array as a tool to identify adaptive mutations, to depict genome organizations, and to identify global regulons by the "ChIP-on-chip" technique will expand our knowledge on *P. aeruginosa *adaptation, evolution and regulatory mechanisms of persistence on a global scale and thus advance the development of effective therapies to overcome persistent disease.

## Background

*Pseudomonas aeruginosa *is a very versatile bacterial organism that has a unique capability to thrive and survive in a great variety of habitats. The bacterium is ubiquitously found in aquatic and terrestic environments and has evolved as an important opportunistic pathogen that causes serious infections in plants, insects and vertebrates [1,2]. In the human host *P. aeruginosa *is mainly a nosocomial pathogen feared for entailing acute pneumonia and sepsis accompanied with very high mortality rates [3,4]. Moreover, *P. aeruginosa *is a dominant bacterial pathogen that causes chronic infections in patients with burns or suffering from cystic fibrosis (CF) [5-7]. Most of the CF patients acquire *P. aeruginosa *from the environment during their early life-time [8] and develop a chronic *P. aeruginosa *lung infection which largely determines the fate and prognosis in these patients. A very characteristic finding is that the same bacterial lineage usually persists for years or even decades in the chronically infected CF lung and cannot be eradicated even by intensified anti-pseudomonas therapy [9]. Thus, in order to overcome chronic persistent disease, novel anti-microbial treatment strategies are desperately needed. For that purpose, it seems to be essential to gain detailed insight into the molecular mechanisms that underlie bacterial adaptation processes to the environment of the cystic fibrosis lung, which results in the evolution of a protected and persistent bacterial population. Recently Smith at al. [10] have conducted a very powerful approach to gain global information on *P. aeruginosa *adaptation to the chronically infected CF lung. They performed whole genome sequencing of an early and a late *P. aeruginosa *CF isolate and could show that many single-base changes accumulated in the late isolate that were obviously advantageous for the life within the host. Intriguingly, these mutations caused loss of functions used by bacteria to invade and injure the host, indicating that they may become a burden once the chronic infection has become established. The work by Smith et al. [10] has opened a window to complex questions of bacterial adaptation and evolution during chronic infections. The great challenge now is to expand the search for adaptive mutations and to validate the findings. Furthermore a more detailed analysis whether evolution produces one adapted strain or whether it produces a diverse community of infecting bacteria is desirable.

In this study we have designed a *P. aeruginosa *Affymetrix custom made "tiling" array (PATA1) composed of approximately 250.000 25-mer oligonucleotides that – due to the large chromosome – depicts approximately 85% of the PAO1 genome. To our knowledge, PATA1 is the first tiling array targeting a complete *Pseudomonas *genome. The probes of a tiling array are more or less equally distributed across the genome instead of using a small subset of probes targeting e.g. ORFs like the Affymetrix PAO1 GeneChip^® ^or other available DNA microarrays. Therefore, tiling arrays produce unbiased data on the whole chromosome. The method has been successfully applied to detect mutations in *Helicobacter pylori *[11]. We evaluated whether with a microarray-hybridisation based method this array is capable of identifying genome variations in *P. aeruginosa *as a cost-effective alterative to whole genome sequencing. By comparative hybridization of chromosomal DNA of a PAO1 and a PA14 strain, we clearly demonstrate that our custom made PATA1 array efficiently detects inter-strain genetic variations even at the level of single nucleotide polymorphisms (SNPs) and can be used as a highly sophisticated tool for the identification of the *P. aeruginosa *genome organization.

## Methods

### Organism & Culturing

The *P. aeruginosa *strains used for the microarray experiments presented in this study were PAO1 (parental strain of the Washington Transposon Mutant Library, [12]) and PA14 (obtained from the Ausubel Lab). *P. aeruginosa *DSM1707 was used as a reference strain to test for the presence of the PA4685 deletion. Cultures were grown in brain-heart infusion (BHI) medium at 37°C in shaking glass flasks or tubes at 180 rpm.

### DNA preparation and Microarray hybridization

Cell samples were harvested from 1 mL of 12 h stationary phase liquid cultures. Genomic DNA was isolated using the DNeasy Blood & Tissue Kit (Qiagen, Hamburg, Germany). Cell lysates were treated with RNase I (Qiagen) to prevent accidental carryover of RNA to the microarray. Genomic DNA was partially digested with DNase I (Amersham Biosciences, Piscataway, NJ) to a fragment size of ~50 – 250 bp, confirmed by gel electrophoresis, and fragments were labeled at the 3'-ends with biotin-ddUTP (Roche Diagnostics, Indianapolis, IN) using Terminal deoxynucleotidyl transferase (Roche).

For each sample 4 – 5 μg of labeled DNA fragments were hybridized to an identical lot of PATA1 array for 16 hours at 50°C.

After hybridization the GeneChips were washed, stained with SA-PE and read using an Affymetrix GeneChip fluidic station and scanner according to Affymetrix standard protocols (Affymetrix, Santa Clara, CA).

### Validation of a 1 kb deletion at PA4685 by PCR

Two sets of primers were designed to test genomic DNA for presence of the 1 kb deletion identified from microarray data. Primers A1 (5'-TCG CAG GTC GAG AGC TAC GT-3') and A2 (5'-ATG CGT CAG CCT CCT GTT GC-3') are placed outside the suspected region while B1 (5'-GCT GCC GGA CCT CAT GCA AT-3') and B2 (5'-TCG CGG TGG CTG ATG TGG TA-3') span a sequence inside this region. DNA polymerase GoTaq (Promega, Madison, WI) was used for PCR.

### Analysis of tiling array data

Analysis of microarray data was performed using the Affymetrix GCOS 1.4 to generate the raw data files (cel data).

The raw data files were further analyzed using 'Tiling Analysis Software' (TAS) version 1.1 by Affymetrix. For probe analysis a bandwidth of 1 (i.e. using only data of single oligonucleotides) was applied. The signal ratio for an oligonucleotide *i *is expressed as *D*_*i *_= log_2_(*S*_*t*,*i*_/*S*_*c*,*i*_), the log_2 _scaled quotient *D*_*i *_of probe signal *S*_*t*,*i *_of the treatment group and probe signal *S*_*c*,*i *_of the control group of arrays.

Integrated Genome Browser (IGB) was used for visualization of signal ratios.

For facilitating array analysis a flexible Java program was developed. Hereby, result text files containing probe signal and p values for the corresponding oligonucleotides are imported and mapped onto known genes and intergenic regions of *P. aeruginosa *PAO1. Analysis results are stored in tab-separated files including significantly detected genes. Beside obtaining gene information from an implemented file, a variable index file can be constructed from an appropriate file presented by the user. This Java program was tested on JRE 1.6.0 and is provided for download (see additional file 1).

## Results

### Design of a whole genome tiling array for *Pseudomonas aeruginosa *PAO1

**Figure 1:**
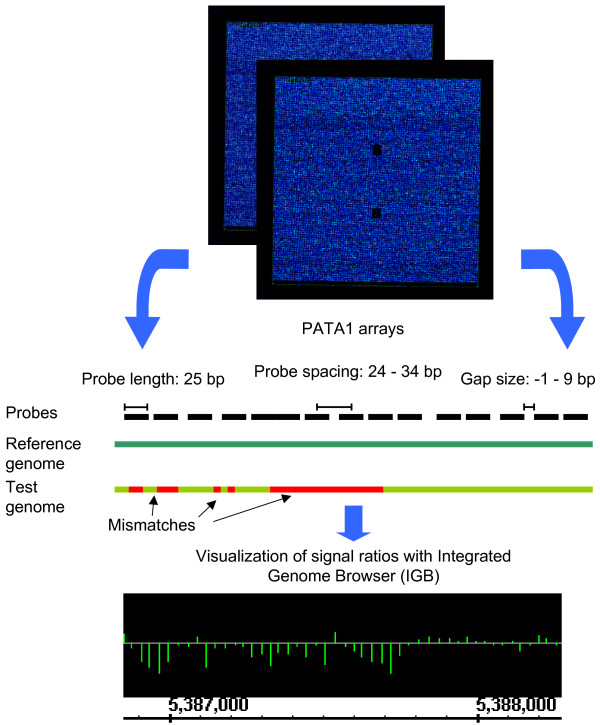
**Array design and comparative hybridization principle**. The *Pseudomonas aeruginosa *Tiling Array (PATA1) includes ~215000 DNA oligonucleotide probes of 25 bp (black bars). Placed with variable probe spacing (24 – 34 bp) these probes cover 85.9% of the PAO1 genome leaving gaps of up to 9 bp (average ~4). For comparative hybridization, labeled DNA fragments of a reference strain (dark green) and the test strain (light green) were hybridized in parallel to separate PATA1 arrays. Signal ratios were calculated for each oligonucleotide as log_2 _ratio of test versus reference signal and can e.g. be visualized by the Integrated Genome Browser (IGB). Genetic variations lead to mismatches of sample and probe DNA and hence to a decrease in signal ratio.

The *P. aeruginosa *Tiling Array PATA1 was designed in cooperation with and manufactured by Affymetrix according to GENECHIP^® ^CUSTOM EXPRESS™ ARRAY DESIGN GUIDE (Affymetrix, Santa Clara, CA). This tiling array was originally developed to screen uncharacterized *P. aeruginosa *strains for genetic differences. The genome sequence of strain PAO1 available from the Pseudomonas Genome Database (www.pseudomonas.com) was used as template for designing perfect match (PM) tiling probes with a density of 28 bp (+/- 5). The standard practice for probe selection is to prune against specific bacterial and species-specific controls. Pruning is a sequence comparison method and increases the quality of the unique probe selected for the design and reduces the risk of cross-hybridization with other sequences (Array Design Guide, Affymetrix, Santa Clara, CA). Sequences used for hard pruning are generally highly repetitive elements, such as alu-like elements, or abundantly expressed RNA, like rRNA. Probes that cross-hybridize to hard pruning sequences were excluded. This resulted in a total of 215169 probes with 25 base pair length (Figure 1). Thus, 85.9% of the whole genome sequence (6,264,404 bp) is present on PATA1 with an average tiling of ~29 bases, hence leaving gaps of 4 bases average length (Figure 1). For two gap regions (1539 and 2907 bases) selected probes were removed as a result of hard pruning with 2 sequences – 16s rRNA and 23s rRNA (ribosomal RNAs). A second round of probe selection was started including only these gap regions and having gaps greater than 56 bp to maximize the coverage of the tiling array while minimizing unspecific hybridization effects. By this we increased the number of probes with 204 additional probes. As negative controls we used 2358 Affymetrix *Arabidopsis *tiling probes as an estimate of background activity.

### In silico comparison of PAO1 array probes with the PA14 genome sequence

In order to estimate how many genetic differences between the two fully sequenced *P. aeruginosa *strains PAO1 and PA14 can theoretically be detected with the array we first identified in an *in silico *approach the best matching sequence of the published PA14 genome for each 25 bp oligonucleotide present on the array. Perfect matches (i.e. 100 per cent sequence identity) of oligonucleotides with the PA14 sequence were identified in an initial run using NCBI's BLAST software [13]. The BLAST search was sufficient for the detection of identical sequences. However, since BLAST uses a heuristic approach that approximates the Smith-Waterman algorithm [14] the alignment is fast but less accurate in finding optimal matches of non-identical sequences. Therefore all oligonucleotides for which BLAST found no perfect match were aligned in a second run using 'JAligner' [15]. JAligner is a freely available JAVA tool that makes use of the original Smith-Waterman algorithm. This algorithm is very slow (analysis of ~48000 oligonucleotides took > 1 week computation time on a standard computer) but always finds the single optimum alignment. Combining the results from both alignments, we could determine the position of the best matching sequence within the PA14 genome, the sequence similarity, i.e. the fraction of nucleotides that is identical in both sequences that are compared, and furthermore the exact positions of mismatches and gaps. Additional to the best matching sequence, BLAST (unlike Jaligner) identified other less than optimal sequences. However, these secondary alignment hits were of much lower quality (i.e. BLAST score) and were therefore not considered for further analysis.

**Figure 2:**
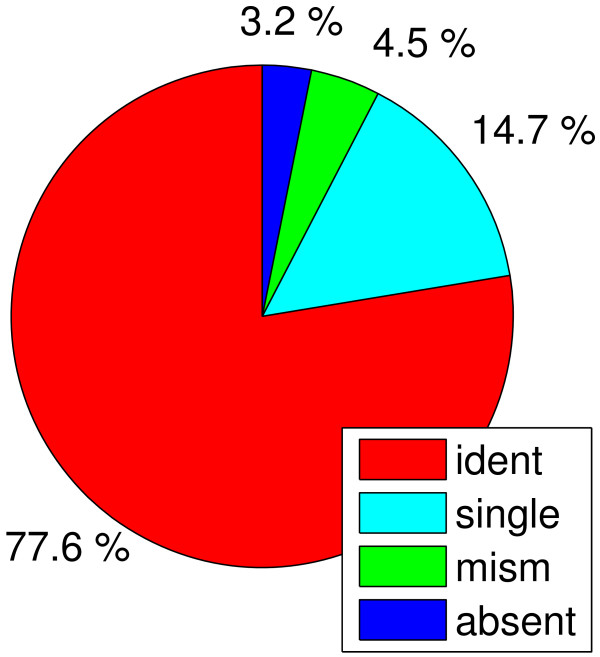
**similarity classes resulting from global alignment of PATA1 oligonucleotides with the PA14 genome**. All oligonucleotides present on PATA1 were classified by their similarity to the best matching hit resulting from an sequence alignment with the PA14 genome sequence. Similarity classes: 'ident' – identical sequence in PAO1 and PA14, 'SNP' – single nucleotide polymorphism (SNPs), 'mism' – oligonucleotides with more than a single mismatch but at least 80% sequence identity and with no more than 2 gaps, 'absent' – less than 80% sequence identity or more than 2 gaps.

The sequence alignment results were subsequently used to classify the oligonucleotides according to sequence similarity (Figure 2). 166985 (77.6%) of all oligonucleotides were found to be identical in sequence to their best matching PA14 sequence (similarity class 'identical'); 31585 (14.7%) showed a single nucleotide polymorphism ('SNP'); 9816 (4.6%) showed more than a single mismatch but still had at least 80% identical nucleotides with not more than 2 gaps ('partial'); and 6783 (3.2%) had a similarity below this threshold and were therefore classified as low matching or 'absent'. It should be noted that, due to the algorithm that was applied, even absent sequences will always result in some match with low similarity that can usually be considered as random.

The core genomes of PA14 and PAO1 show a high synteny (i.e. orthologous genes are located in the same order in both genomes) with the exception of a large genomic inversion between two of the four ribosomal gene clusters [16,17]. This inversion is also reflected in the alignment data of the PATA1 oligonucleotides and the PA14 genome: for a major part of the genome the best matching sequences in PA14 were found in reversed order and on the opposite strand. The best matches of 'absent' oligonucleotides where distributed over the whole genome of PA14, which indicates that these hits are most probably random hits.

**Figure 3:**
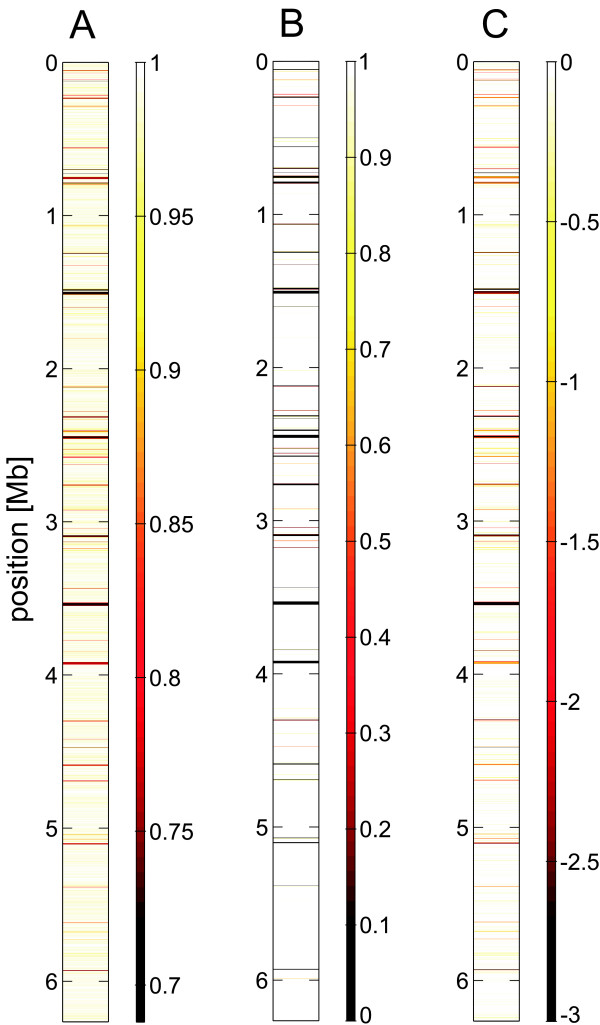
**identification of PAO1 specific regions**. These 'chromosomal maps' display the whole PAO1 genome as a vertical strip with horizontal bands each representing a region of 1 kb at the indicated position on the chromosome with a color coded value (see color bars to the right of each map). A) Sequence similarity of PATA1 oligonucleotides and the PA14 genome. Values indicate the fraction of nucleotides that were identical in the probe oligonucleotides and the best matching alignment in PA14. B) PAO1 specific regions. The actual value indicates the fraction of DNA which is common to PAO1 and PA14 as identified in a previous ORF-by-ORF alignment [16]. C) Signal ratio (log_2_) of a comparative hybridization of PA14 DNA relative to PAO1 DNA, calculated from data of single arrays for PA14 and PAO1. Values exceeding the presented interval (-3 to 0) were cut off to keep the color scale in the same range as in A) and B).

In Figure 3A we depicted a 'chromosomal map' of the PAO1 chromosome displayed as horizontal bands each one representing a region ('window') of 1 kb length. Regions of low sequence similarity in comparison to the PA14 genome, i.e. regions specific to PAO1, appear as darker bands (red or black) in the maps, whereas regions of high average sequence similarity are white. We furthermore compared this chromosomal map with that generated on the basis of a previously published work [16] where the authors identified 75 PAO1 specific regions that were larger than 10 bp by an ORF by ORF alignment of the PAO1 and PA14 sequence (Figure 3B). Both analyses generate a very similar pattern suggesting that this pattern could function as a unique 'genomic fingerprint' for strain PA14. Recently, the genome-wide comparison of five *P. aeruginosa *strains identified regions showing significant inter-strain variation [18]. 23 *regions of genomic plasticity *(RGP) containing variations or insertions that are specific to PAO1 as compared to PA14 were identified and all of them are also represented in our genomic fingerprint (not shown).

### Comparative hybridization of the PAO1 and PA14 genomes

**Figure 4:**
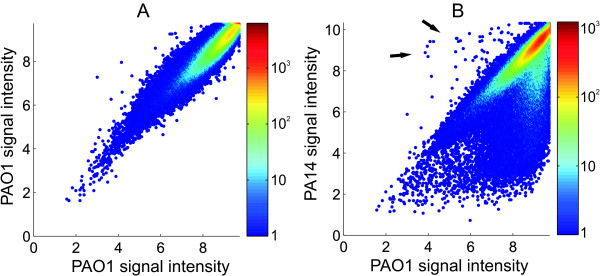
**hybridization ratio variation**. Scatterplots showing the difference in hybridization signal between two independent arrays. Data points represent normalized signal intensities (log_2 _scale) of oligonucleotides. Colors indicate the number of data points that have been grouped for a better visualization (see color bar for scale). A) Intra strain variation of signal intensity. Two independent samples of PAO1 DNA were comparatively hybridized. Signal intensities show high correlation (R = 0.935) with most data points being close to the diagonal. B) Variation of signal intensity between PA14 and PAO1. Two single arrays were comparatively hybridized with PA14 DNA and PAO1 DNA, respectively. The large cloud of data points below the diagonal indicates sequence variations that lead to decreased signal ratios for the affected oligonucleotide probes. Data points significantly above the diagonal (arrows) are due to a 1 kb deletion in the reference PAO1 strain (for details see Figure 7 and text). Note that a small proportion (~2%) of signals shows saturation effects leading to the strict edges of the data clouds.

To analyze the potential of PATA1 to detect genetic differences, we comparatively hybridized DNA of PAO1 and PA14 on the array. The probe signals (measured as absolute signal intensities for each oligonucleotide probe) of different oligonucleotides on a single array showed large variation (~ by a factor of 10) for both the PA14 and the reference PAO1 DNA, presumably due to different binding affinities that depend more or less directly on the oligonucleotide sequence. However, a comparison of two independent PAO1 arrays demonstrated that the variation within one microarray was reproducible and variation between data for the same probe on different arrays was considerably low (Figure 4A). A pair wise comparison of 6 independent PAO1 arrays showed high correlation of corresponding probe signals with an average correlation coefficient of R = 0.960 (for 6 PA14 arrays: R = 0.966). When hybridizing PA14 chromosomal DNA to the array, we observed a large number of decreased probe signals putatively indicating genetic variations (Figure 4B). The signal ratios (log_2 _scale ratios of PA14 and PAO1 probe signals) revealed the same unique pattern of PAO1 specific regions, that was already identified in the *in silico *sequence alignment (Figure 3C). Again, PAO1 strain specific regions appeared as darker bands on a bright background of regions with high sequence similarity. The highly reproducible fingerprint like pattern of sequence similarity on the chromosomal scale does not only provide a tool for strain identification, but also allows detailed determination of the genomic structure, e.g. presence and location of genomic islands and strain specific regions.

As opposed to the identification of the presence or absence of strain specific regions, the detection of inter-strain variations at the level of single oligonucleotides is less clear cut. Signal ratios of variant and unchanged regions are overlapping as can be observed in the scatter plot of the comparative hybridization of PAO1 and PA14 (Figure 4B). Therefore, any threshold applied to filter out variant signal ratios will lead to disregard of some variant regions because their signal ratio is not changed enough (false negatives) and on the other hand detection of unchanged regions that show for some reason a variation to a lowered signal ratio (false positives). The quality of the set of oligonucleotides that is detected as variant by applying a certain threshold can be expressed in terms of *sensitivity *and *specificity *of the detection. We defined an oligonucleotide *i *as 'detected' (i.e. showing a reduced signal ratio that supposedly indicates a sequence variation), if its signal ratio *D*_*i *_was below *D*_*th*_, the signal ratio threshold (*D*_*i *_≤ *D*_*th*_). The *sensitivity *of detection was defined as

(1) $$sens(D_{th})=\frac{N_{\mathrm{var}}(D_{th})}{N_{\mathrm{var}}^{0}}$$

where *D*_*th *_denotes the signal ratio threshold, *N*_var_(*D*_*th*_) the number of oligonucleotides with sequence variations (as identified *in silico*) that showed a signal ratio *D*_*i *_≤ *D*_*th *_(i.e. true positive detections), and $N_{\mathrm{var}}^{0}$ the total number of variant oligonucleotides. Thus, sensitivity describes how many oligonucleotides that are different to the PA14 sequence actually are detected upon application of a certain *D*_*th*_.

Furthermore, we defined *specificity *as

(2) $$spec(D_{th})=\frac{N_{\mathrm{var}}(D_{th})}{N_{total}(D_{th})}$$

where *N*_*total*_(*D*_*th*_) is the number of all oligonucleotides with *D*_*i *_≤ *D*_*th *_(including false positives). Hence, specificity describes the fraction of correct detections (true positives) among all detections.

**Figure 5:**
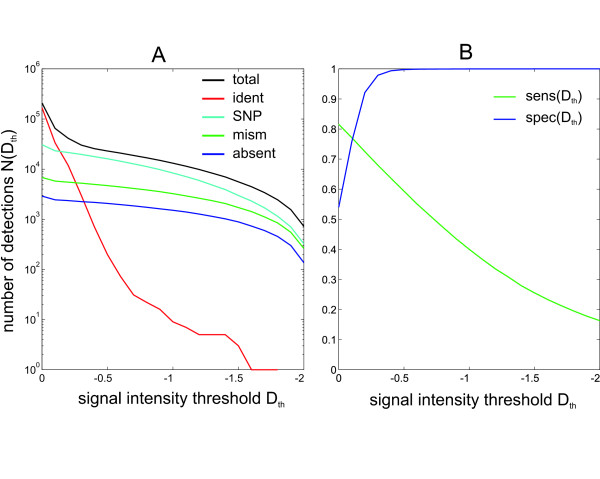
**detection of variations – sensitivity and specificity**. Detection of variant oligonucleotides from a comparative hybridization of PA14 and PAO1 DNA. For each strain two independent arrays were used. A) Detection of certain variations by similarity class (Figure 2) for different values of signal ratio threshold *D*_*th*_. More restrictive (i.e. more negative) threshold values reduce the number of overall detections (*total*) but false positive detections (*ident*) are affected more than the others. Each curve represents the number of oligonucleotides that are detected (i.e. that show a signal ratio *D*_*i *_≤ **D**_*th*_) using a given *D*_*th*_. *total *(black curve) indicates the sum of all detections, while *ident *(red), *SNP *(cyan), *mismatch *(green) and *absent *(blue) refer to the detection numbers of the respective similarity classes as described in Figure 2. B) Sensitivity and specificity. Sensitivity is defined as the fraction of variant oligonucleotides that are actually detected using a certain threshold *D*_*th *_(Equation 1). Specificity is defined as the ratio of false positive detections (such of 'identical' oligonucleotides) among all detections (Equation 2). The dashed vertical line indicates a threshold *D*_*th *_= -0.5 as it is used in the text.

In order to find a threshold optimizing sensitivity and specificity we determined the number of detected oligonucleotides for a range of *D*_*th *_values based on a data set obtained from comparative hybridization of 2 independent arrays for both PA14 and PAO1 (Figure 5). Application of a more restrictive, i.e. more negative, *D*_*th *_generally reduced the detection, as reflected by the detection counts (Figure 5A) and thus the sensitivity (Fig 5B). E.g., more than 10^4 ^variant oligonucleotides were detected using *D*_*th *_= -1 while only ~10 false positives remained (Figure 5A). However, this high specificity of ~0.9996 (Figure 5B) lead to a drop in sensitivity to 0.40, meaning 60% true hits were missed in this case. A significantly higher sensitivity of 0.59 resulted for *D*_*th *_= -0.5 while specificity was still high (0.997). Sensitivity further increased with a less restrictive *D*_*th *_but at the cost of a strong decrease in specificity (Figure 5B).

Remarkably, such high sensitivities could already be achieved by comparative hybridization of only one single array for each PAO1 and PA14, though the values showed variation when different arrays were compared (Table 1). Including more arrays in an analysis (which would normally be avoided because of the cost factor) did not increase the sensitivity. Specificity, on the other hand, increased with larger sets of arrays (Table 1) indicating that the observed variation in sensitivity is caused by falsely positive detection of oligonucleotides, which occurs randomly. When attempting to identify genetic variations in uncharacterized strains, it seems therefore reasonable to use even only one array for both the test and reference strain as a first scan for putative variations. More arrays could then be used to increase specificity as a validation of results, although this could as well be achieved by less sophisticated (and costly) methods such as sequencing of a putative variant gene.

**Table 1:** Influence of the size of data sets on the sensitivity and specificity of different data sets

			sensitivity			specificity
treatment group size^a^	control group size^a^	total	SNP	mismatch	absent	total
1	1	**0.585**	0.481	0.740	0.848	**0.989**
1	1	**0.600**	0.500	0.747	0.860	**0.967**
1	1	**0.588**	0.479	0.751	0.866	**0.998**
1	1	**0.603**	0.497	0.759	0.874	**0.989**
2	2	**0.593**	0.488	0.750	0.863	**0.997**
4	4	**0.590**	0.483	0.752	0.866	**0.998**
6	6	**0.572**	0.459	0.742	0.863	**0.999**

^a ^The four analyses of 2 × 1 array are the four possible combinations of arrays that were used in the 2 × 2 analysis. The 2 × 4 and 2 × 6 analyses contain the above stated microarrays plus additional ones.

### Detection of variations at the gene level

Since the Tiling Analysis Software (TAS) provided by Affymetrix can process the results of a comparative hybridization only at the level of oligonucleotides and a user usually might be more interested in the genes that are affected by the detected variations, we also developed a Java software tool to translate these results to the level of genes. The software reads the output data produced by TAS and applies a signal threshold *D*_*th *_to filter out candidate variant oligonucleotides. The oligonucleotide positions are then used to map these detections to the corresponding genes or intergenic regions. As an example, Table 2 shows the results of both the sequence alignment and a comparative hybridization analyzed with TAS and our JAVA tool for the gene clusters containing *wbp *and related genes that are involved in the synthesis of lipopolysaccharides (LPS). PAO1 contains two clusters of *wbp *genes, one of which (PA3141-PA3160) is specific to PAO1 [18] while the other (PA5447-PA5452) is present in both PAO1 and PA14. In case of the specific genes of the PA3141-PA3160 cluster, most of the oligonucleotides covering those genes belonged to the 'mism' or 'absent' similarity class in the *in silico *alignment. The comparative hybridization also detected most of the oligonucleotides of the specific genes as absent, which is also reflected by the high sensitivity close to 100%. The other gene cluster showed a high sequence similarity in both strains with a few SNPs present in each gene (Table 2). Detection of those SNPs showed sensitivities around 40 – 60% corresponding to the global average for SNPs.

**Table 2:** *In silico *comparison and comparative hybridization results for the two *wbp *gene clusters of PAO1

**gene information**			**in silico analysis**						**comparative hybridization**	
			count of similarity classes^a^							
PAO1 locus ID	PA14 locus ID	gene name	ident.	SNP	mism.	absent	total	average sequence similarity	detected oligos^b^	sensitivity^b^
PA3141	PA14_23470	*wbpM*	54	7	4	5	70	0.97	12	0.75
PA3142*	absent		4	3	4	1	12	0.93	7	0.88
PA3143*	absent		4	4	8	3	19	0.90	11	0.73
PA3144*	absent		1	0	4	0	5	0.91	4	1.00
PA3145*	PA14_23460	*wbpL*	0	0	3	33	36	0.71	35	0.97
PA3146*	PA14_23450	*wbpK*	0	0	6	28	34	0.71	33	0.97
PA3147*	absent	*wbpJ*	0	0	6	37	43	0.72	43	1.00
PA3148*	absent	*wbpI*	0	0	7	30	37	0.74	37	1.00
PA3149*	absent	*wbpH*	0	0	6	33	39	0.72	39	1.00
PA3150*	absent	*wbpG*	0	0	3	36	39	0.70	39	1.00
PA3151*	absent	*hisF2*	0	0	3	24	27	0.69	26	0.96
PA3152*	absent	*hisH2*	0	0	1	21	22	0.70	22	1.00
PA3153*	absent	*wzx*	0	0	1	43	44	0.71	43	0.98
PA3154*	absent	*wzy*	0	0	1	45	46	0.70	46	1.00
PA3155*	absent	*wbpE*	0	0	5	33	38	0.72	38	1.00
PA3156*	absent	*wbpD*	0	0	4	17	21	0.72	20	0.95
PA3157*	absent		0	0	14	51	65	0.73	64	0.98
PA3158*	absent	*wbpB*	0	0	7	26	33	0.74	33	1.00
PA3159*	absent	*wbpA*	0	0	14	32	46	0.73	46	1.00
PA3160	PA14_23360	*wzz*	0	0	5	31	36	0.71	36	1.00
PA3161	PA14_23340	*himD*	8	1	1	0	10	0.99	1	0.50
										
PA5447	PA14_71910	*wbpZ*	35	4	1	0	40	0.99	5	1.00
PA5448	PA14_71920	*wbpY*	34	5	0	0	39	0.99	2	0.40
PA5449	PA14_71930	*wbpX*	40	8	1	0	49	0.99	4	0.44
PA5450	PA14_71940	*wzt*	40	5	0	0	45	1.00	3	0.60
PA5451	PA14_71960	*wzm*	24	5	0	0	29	0.99	3	0.60
PA5452	PA14_71970	*wbpW*	42	6	1	0	49	0.99	4	0.57

* genes belonging to the PAO1 specific region.

^a ^see Figure 2 for a definition of the similarity classes.

^b^determined for *D*_*th *_= -0.5.

### Detection of mismatches is affected by mismatch position and the type of nucleotide substitution

**Figure 6:**
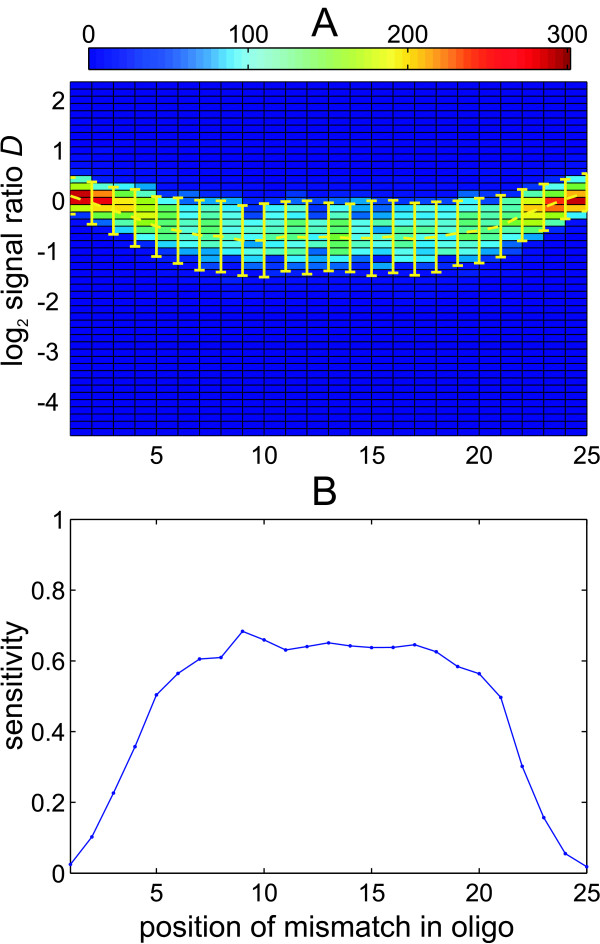
**mismatches placed at the margins of oligonucleotides are hard to detect**. A) 3D histogram grouping oligonucleotides according to mismatch location and signal ratio (log_2 _scaled). Only oligonucleotides covering a single nucleotide polymorphism (SNP) with the corresponding PA14 sequence are included. Each rectangle represents the group of all oligonucleotides with a SNP at the specified position (x-axis) showing the specified hybridization ratio (y-axis). The dashed yellow line indicates the average hybridization ratio of all oligonucleotides with common mismatch location. Error bars indicate one standard deviation. B) Sensitivity (see Figure 5) for detection of oligonucleotides covering a SNP (*D*_*th *_= -0.5).

The detection of mismatches by comparative hybridization relies on the decreased formation of probe/sample DNA duplexes. It has been show previously that the hybridization of DNA fragments on surface tethered probes is affected by various factors, such as the position of the mismatch within the duplex and the stacking energy at this position [19], or surface properties of the array [20]. In our *in silico *analysis 31585 oligonucleotides with single nucleotides polymorphisms (SNPs) could be identified ('SNP', Figure 2), enabling a detailed analysis of SNP detection from our experimental data. Grouping all SNP oligonucleotides according to the location of the mismatch demonstrated that mismatches at marginal positions mostly lead to a much smaller decrease of signal ratio as compared to mismatches located at a more central position (Figure 6A). The average signal ratio for a mismatch at a central position was about -0.7 and approached zero at the positions close to either of both ends, which also severely affected the sensitivity (Figure 6B). This significant drop in sensitivity can be observed at the 4 outermost positions of either side (1–4, 22–25), while sensitivity was relatively similar (around 60%) for the central 17 positions.

To analyze the influence of the type of nucleotide present in the mismatching position we grouped all SNPs according to the alleles present in the PATA1 probes and the PA14 sequence, respectively. This analysis revealed a marked bias in the median (average) signal ratios of certain SNPs (Table 3). The median signal ratios differed from -0.57 for SNPs where a 'T' within the probe corresponded with a 'C' within the PA14 sequence (PAO1-T/PA14-C) down to only -0.24 for SNPs with alleles PAO1-C/PA14-A. Most notably, this effect seemed to be asymmetric, since the median signal ratios for opposite SNPs, like e.g. PAO1-T/PA14-A and PAO1-A/PAO1-T, showed large differences in most cases (Table 3).

**Table 3:** Median signal ratio of SNPs grouped by the bases present in both alleles.

		**corresponding PA14 sequence**^a^			
		**A**	**C**	**G**	**T**
**PATA1 probe (PAO1)**^a^	**A**	-	-0.50	-0.51	-0.41
			(1031)	(6807)	(326)
	**C**	-0.24	-	-0.35	-0.30
		(670)		(1283)	(5741)
	**G**	-0.32	-0.43	-	-0.33
		(5874)	(1309)		(687)
	**T**	-0.49	-0.57	-0.45	-
		(331)	(6574)	(952)	

Numbers in brackets indicate the abundance of each SNP in our data.

^a ^mismatching nucleotide in the oligonucleotide probe and in the PA14 sequence, respectively.

Furthermore, grouping the different SNPs according to the category of the nucleotide substitution revealed that the bias observed in our data strongly depended on the 'interaction type' of the nucleotides present in either allele. The nucleotides G and C can be categorized as showing 'strong interaction' (common symbol in IUPAC format: 'S') because they can form three hydrogen bonds in contrast to A and T which show only weak interaction ('W'). While any SNP constitutes a mispairing and thus no regular H bonds are formed, the median signal ratio was different for different combinations of interaction types (S or W) in the probe and sample alleles (Table 4). The strongest decrease in signal ratio (-0.53) was observed for SNPs, where an S nucleotide in the PA14 sample DNA corresponded to W in the PAO1 probe (PAO1-W/PA14-S). On the other hand, SNPs with PAO1-S/PA14-W showed only a median signal ratio of -0.30. This asymmetry cannot be explained by the sequence, which is the same except for the mismatch itself, and probably results from the surface fixation of the probe. It has been shown previously that surface effects affect duplex formation [19,20].

**Table 4:** Signal ratios of SNPs are biased towards the interaction type (strong or weak) of the bases present in the probe and sample alleles.

**PAO1 allele (probe)**^a^	**PA14 allele (sample)**^a^	**abundance**	**median signal ratio**	**sensitivity**^b^
W	S	15364	-0.53	52.1%
W	W	657	-0.46	47.5%
S	S	2592	-0.38	43.7%
S	W	12972	-0.30	39.1%

Σ		31585	-0.43	45.9%

^a ^W – IUPAC format for bases with weak interaction (A, T), S – strong interaction (G, C).

^b ^determined for *D*_*th *_= -0.5.

The marked differences in abundance of SNP types (Table 3) result mostly from a biased *transition*/*transversion *ratio. According to our data, transitional substitutions, i.e. mutations from purine (G, A) to purine or pyrimidine (C, T) to pyrimidine, occur about 4 times as often as transversions (purine to pyrimidine or vice versa) (Table 5). However, the signal ratios of transversions and transitions did not show any significant bias.

**Table 5:** Signal ratios of SNPs are independent from the chemical class (pyrimidine or purine) of the bases present in the probe and sample alleles.

	**PAO1 allele (probe)**^**a**^	**PA14 allele (sample)**^**a**^	**abundance**	**median signal ratio**	**sensitivity**^**b**^
transitions	Y	Y	12315	-0.44	46.9%
	R	R	12681	-0.43	45.8%
transversions	R	Y	3353	-0.44	46.9%
	Y	R	3236	-0.37	43.0%

	Σ		31585	-0.43	45.9%

^a ^Y – IUPAC format for pyrimidine bases (C, T), R – purine bases (A, G).

^b ^determined for *D*_*th *_= -0.5.

We also examined the influence on the signal ratio of the nucleotides that are direct neighbors of the mismatch. There are 64 possible trinucleotides formed by the SNP nucleotide and its direct neighbors and 3 different alleles forming a mismatch at the central position with these trinucleotides. The results for each of these 192 different combinations is provided in additional file 2. The signal ratio is on average less negative if a SNP is flanked by strongly interacting bases (G or C) as summarized in Table 6. Therefore, such SNPs are more difficult to detect as indicated by the lower sensitivity.

**Table 6:** Signal ratios of SNPs are influenced by interaction of neighboring nucleotides

**neighboring nucleotides**	**abundance**	**median signal ratio**	**sensitivity**^**b**^
W-W^a^	3223	-0.77	63.2%
W-S or S-W	14599	-0.54	52.5%
S-S	11123	-0.39	42.7%
marginal^c^	2640	0.07	2.2%

	31585	-0.43	45.9%

^a ^W – IUPAC format bases A, T showing weak interaction, S – IUPAC for G, C showing strong interaction.

^b ^determined for *D*_*th *_= -0.5.

^c ^SNPs in the marginal position 1 or 25 that don't have two neighboring nucleotides (see also Figure 6).

### Detection of a 1 kb deletion in the Washington Genome Center PAO1 strain

**Figure 7:**
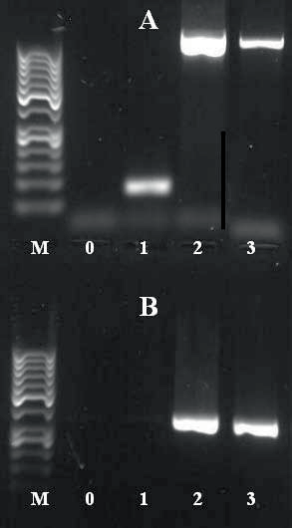
**Identification of a 1 kb deletion in MPAO1**. Gelelectrophoresis after PCR Amplification of the PA4685 region using primer pair A1/A2 ('A') or B1/B2 ('B'). M – GeneRuler 50 bp (Fermentas, Hanover, MD), 0 – control PCR lacking chromosomal DNA, 1 – PAO1 Washington Genome Center, 2 – PA14, 3 – PAO1 DSM1707.

Since PA14 is closely related to PAO1 and more than 75% of the PATA1 oligonucleotides correspond to identical sequences in PA14, the comparative hybridization of PAO1 and PA14 DNA should allow not only the detection of variations in PA14 as opposed to PAO1 but also of variations in the chromosomal DNA of PAO1 as opposed to the hypothetical PAO1 sequence which was used for designing the PATA1 oligonucleotide probes. In contrast to the above described variations in PA14, those PAO1 variations should appear as an increase in probe signal. Indeed, we identified a whole set of 35 oligonucleotides with signal ratios between 0.97 (~2 fold increase) and 5.17 (~36 fold increase), which all together covered parts of the two hypothetical ORFs PA4684 and PA4685. Since the increased signal ratio resulted from a decrease in the PAO1 probe signals while PA14 probe signals were unaffected, we postulated a partial deletion of these two genes in the Washington PAO1 strain to be the cause of the observation. This hypothesis was tested by PCR amplification of (i) the whole region including short parts of the flanking sequence (primers A1, A2) and (ii) a shorter sequence inside the hypothetical deletion (primers B1, B2). The size of this deletion could be predicted to be between 1005 – 1020 bp considering the location to the unaffected oligonucleotide that were closest to that region. The PCR results using PAO1 and PA14 chromosomal DNA, respectively, clearly indicated a size shift of the expected band for primer pair A1/A2 of ~1000 bp which perfectly fits the prediction (Figure 7) and with primers B1/B2 no PCR product was observed for PAO1. As an alternative to the Washington Genome Center PAO1 strain that was used for microarray hybridization, we also tested PAO1 DSM1707 which like PA14 resulted in PCR products corresponding to the published sequence of PA4684/4685. These results clearly indicate a genetic difference between different strains of PAO1.

## Discussion

In this study we have successfully designed and evaluated a tiling microarray which targets the whole chromosome of the facultative pathogen *P. aeruginosa *PAO1. Given PAO1's large genome size and a limited construction capacity, one microarray depicted the genome with 25-base-long oligonucleotides tiled with an average 29 base pair spacing which corresponded to an average gap between the oligonucleotides of 4 base pairs.

The alignment of all PAO1 derived 25 base pair sequences present on the *P. aeruginosa *microarray with the published genome sequence of strain PA14 identified regions specific to PAO1 that largely correspond the results of previous studies based on ORF-by-ORF alignment [16]. Experimental data from comparative hybridization of PA14 and PAO1 DNA on the PATA1 chip reproduced this pattern at the chromosomal scale (Figure 3), indicating that genetic differences affecting multiple consecutive oligonucleotides on the array can be detected at very high sensitivity and specificity, even when data were obtained from only one comparative hybridization.

The comparative hybridization of a reference PAO1 obtained from the Washington Genome Centre and the PA14 strain, furthermore lead to the identification of a 1 kb deletion in the Washington PAO1 strain, as compared to the published PAO1 sequence. This deletion affected part of the PA4684 and almost the whole PA4685 gene and explains why in the Washington transposon mutant collection no insertions within the PA4685 gene have been found.

Whereas genetic differences affecting multiple consecutive oligonucleotides on the array can easily be detected, we were also interested in the performance of the *P. aeruginosa *genome array in the identification of single-base pair changes. A similar microarray has previously been developed and successfully applied for the identification of even single-point mutations leading to metronidazole resistance in *H. pylori *strains [11]. To evaluate the performance of the PATA1 array we compared an *in silico *alignment with experimental comparative hybridization data in great detail and identified three factors influencing the sensitivity in the detection of single nucleotide mismatches: i) positional bias – mismatches located at the 4 marginal positions from either side of the oligonucleotide were detected with a much lower sensitivity than mismatches at central locations, leaving a core region of 17 nucleotides; ii) interaction sites bias – sensitivity was decreased for mismatches where a C or G (3 possible H bonds) on the PAO1 probe sequence was corresponding to an A or T (2 possible H bonds) in the PA14 sample sequence and vice versa; and iii) neighbor bias – sensitivity was increased if the nucleotides neighboring the mismatch showed weaker binding, i.e. fewer H bonds (A or T), and vice versa.

Because the sensitivity towards point mutations was significantly reduced at the outer 4 positions at either end of an oligonucleotide, the core regions of all oligonucleotides covered only ~58% of the PAO1 chromosome effectively. This limitation leaves space for a next microarray generation which should depict the whole genome with 25-base-long oligonucleotides tiled with a 16 base pair or less spacing (corresponding to more than 400.000 oligonucleotides). However, despite this low effective coverage, about 50% of all single nucleotide polymorphisms (SNPs) theoretically covered by the PATA1 array could be detected in a comparative hybridization of PAO1 and PA14 genomes using a threshold hybridization ratio of *D*_*th *_= -0.5. Variations larger than SNPs were detected with a much higher sensitivity (up to 85%) leading to an overall detection of about 60% of all theoretical variations with high specificity.

Our results clearly indicate that the microarray hybridization presented in this study represents a very robust method to screen whole sets of *P. aeruginosa *strains bearing unknown genetic variations. One attractive future application of these microarrays could be to identify and depict the *P. aeruginosa *genome organization of clinical strains. Most of the CF patients acquire *P. aeruginosa *from the environment early in life and suffer from transient airway infections with diverse strains of *P*. *aeruginosa*, whereas at a later stage the patients become permanently colonized with one or few *P. aeruginosa *clones [8]. Although diverse environmental *P. aeruginosa *isolates cause chronic infections in the CF lung, it has recently been reported that there are dominant clones in the environment and disease and that individual clones prefer a specific repertoire of accessory segments [21]. In contrast to the core genome, which is mostly shared by all *P. aeruginosa *strains, the accessory genome is highly strain specific [18].

The microarray could be used as a highly sophisticated fingerprinting method to significantly advance the question of whether there are specific genome organizations or certain genetic elements in clinical strains that are more frequently associated with chronic disease and with adverse clinical outcome. These traits may serve as important prognostic markers and may be targets of future drugs designed specifically for action against chronic infections. The recent development of next generation microarrays will offer the opportunity to include strain specific markers (pathogenicity islands) of common *P. aeruginosa *clones.

Furthermore arrays that cover the whole *P. aeruginosa *genome (with tighter tiling) might serve as an alternative cost-effective method and a clear alternative to whole genome sequencing strategies for the identification of genetic variations. The availability of an easy to perform mutation discovery method in *P. aeruginosa *will make a very important contribution and will significantly advance the field of patho-adaptive *P. aeruginosa *evolution during the chronic infection process. *P. aeruginosa *undergoes an intense genetic adaptation processes during the establishment of chronic pulmonary infections in the CF lung in which (mainly single-base pair change) mutations leading to the loss of function of multiple genes are positively selected [10]. This adaptive behavior seems to be crucial in the development of chronic persistent disease, were *P. aeruginosa *resides in a protected niche within biofilms and hides from the host immune responses. A detailed knowledge on general patho-adaptive mutations will lead to the identification of infection relevant bacterial traits which might be very interesting targets for the development of alternative treatment strategies effective against chronic persistent diseases.

Moreover, since the *P. aeruginosa *microarray presented in this study depicts the whole PAO1 genome including the intergenic regions, the array could also serve as a valuable tool to identify binding sites of transcriptional regulators via the "ChIP-on-chip" technique. ChIP-on-chip (Chromatine Immuno Precipitation), is a genome-wide location analysis and a technique for isolation and identification of the DNA sequences occupied by specific DNA binding proteins in cells [22]. More than 9% of the open reading frames in *P. aeruginosa *PAO1 encode for (putative) transcriptional regulators or two-component systems which facilitate efficient adaptation to varied habitats [17]. The identification of the transcriptional regulons involved in the regulation of functions required for bacterial persistence could significantly advance knowledge on specific *P. aeruginosa *adaptation to the environment of the CF lung.

## Conclusion

Chronic *P. aeruginosa *infections remain a major challenge for the medical profession, because even intensified antimicrobial therapy is usually not sufficient to eradicate e.g. persistent infections of the CF lung. Thus, novel anti-*Pseudomonas *treatment strategies that target the functions required for bacterial persistence are desperately needed. In this study we have developed a robust microarray-hybridization based method that can be applied i) to identify patho-adaptive mutations that facilitate *P. aeruginosa *persistence during chronic infection, ii) as a highly sophisticated bacterial fingerprinting method that may help to identify specific clones or genetic elements that are associated with adverse clinical outcomes, and iii) to identify global regulons by the "ChIP-on-chip" technique that play major roles in the regulation of virulence. These applications should significantly expand our knowledge on bacterial adaptation, evolution and regulatory mechanisms of persistence on a global scale and thus should advance the development of effective antibacterial treatment strategies to overcome persistent disease.

## Competing interests

The authors declare that they have no competing interests.

## Authors' contributions

FB, RG and SH designed the array in cooperation with Affymetrix. RG performed array hybridization and data acquisition. AD performed the *in silico *alignment and DNA preparation and analyzed the results. AD and CP designed software tools for analysis beyond the scope of the Affymetrix software. AD and SH wrote the manuscript. All authors have read and approved the final manuscript.

## Supplementary Material

Additional file 1 **Java program used for analysis of the PATA1 data.** We developed a Java program for post-processing of probe signal and p value data produced by the TAS software (see 'Methods'). The zip file contains a jar archive including program binaries and source code, a sample data set and a readme file with brief instructions for installation and usage. Click here for file

Additional file 2 **Influence of neighbouring nucleotides on sensitivity towards SNPs.** For any SNP as identified in the sequence alignment of PATA oligonucleotide probes were grouped by the nucleotides present in both alleles and at the directly neighbouring positions. E.g. 'CGA - T' indicates a mismatch with 'C*G*A' in PAO1 and 'C*T*A' in PA14. For each of all 192 possible permutations, 'abundance' indicates how many oligonucleotides showed this particular combination as identified by sequence alignment and 'sensitivity' indicates the fraction of these oligonucleotides that were detected as variant using a threshold signal ratio *D*_*th *_= -0.5. Click here for file
